# Rational Practices to Manage Boll Weevils Colonization and Population Growth on Family Farms in the Semiárido Region of Brazil

**DOI:** 10.3390/insects5040818

**Published:** 2014-10-31

**Authors:** Robério C. S. Neves, Felipe Colares, Jorge B. Torres, Roberta L. Santos, Cristina S. Bastos

**Affiliations:** 1 Departamento de Agronomia-Entomologia, Universidade Federal Rural de Pernambuco. Rua Dom Manoel de Medeiros, s/n, Dois Irmãos, Recife, 52171-900, Brazil; E-Mails: roberiocneves@yahoo.com.br (R.C.S.N.); fcolaresbatista@yahoo.com.br (F.C.); 2 Departamento de Fitotecnia, Universidade Federal de Viçosa. Av. Peter Henry Rolfs, s/n, Campus Universitário, Viçosa, 36570-000, Brazil; E-Mail: roberta.leme@ufv.br; 3 Instituto Central de Ciências Ala Sul (ICC-Sul), Faculdade de Agronomia e Medicina Veterinária (FAV), Universidade de Brasília (UnB), Brasília, 70910-900, Brazil; E-Mail: cschetino@unb.br

**Keywords:** integrated pest management, physical control, cultural control, kaolin

## Abstract

Because boll weevil, *Anthonomus grandis* Boh. develops partially protected inside cotton fruiting structures, once they become established in a field, they are difficult to control, even with nearly continuous insecticide spray. During two cotton-growing seasons in the Semiárido region of Pernambuco State, Brazil, we tested the use of kaolin sprays to disrupt plant colonization through visual cue interference, combined with removal of fallen fruiting bodies to restrain boll weevil population growth after colonization. Kaolin spray under non-choice trials resulted in 2.2×, 4.4×, and 8.6× fewer weevils, oviposition and feeding punctures on kaolin-treated plants, respectively, despite demonstrating no statistical differences for colonization and population growth. Early season sprays in 2010 occurred during a period of rainfall, and hence, under our fixed spraying schedule no significant differences in boll weevil colonization were detected. In 2011, when kaolin sprays were not washed out by rain, delayed boll weevil colonization and reduction on attacked fruiting bodies were observed in eight out of 12 evaluations, and kaolin-treated plots had 2.7× fewer damaged fruiting bodies compared to untreated plots. Adoption of simple measures such as removal of fallen fruiting bodies and prompt reapplication of kaolin sprays after rainfall show promise in reducing boll weevil infestation.

## 1. Introduction

The impact of various arthropod pest species upon cotton production is considerable, especially that of the boll weevil, *Anthonomus grandis*. Boll weevils typically reduce cotton yields by 54%–87% in the Semiárido region of Paraiba and Pernambuco States, Brazil [1]. Weevil adults colonize cotton fields as early as the first appearance of flower buds (cotton squaring) and continue to feed and develop inside fruiting structures throughout the crop’s phenology. Upon squares, they feed and oviposit [2,3,4]. Oviposition in these cotton squares induces abscission before bolls develop, causing direct yield loss [5,6]. Besides natural shedding, fallen attacked squares allow larvae and pupae to fulfill development in their interior, resulting in faster boll weevil population growth [7]. Continuous fruit production by cotton plants results in late infestations as multiple generations attack developing bolls that, depending on age, may fall or remain on the plant without abscission [8].

Boll weevil immature stages exhibit low natural mortality from natural enemies [9,10] and are difficult to control with insecticides, because they are partially protected inside cotton fruit bodies. Current insecticide-based practices require 5–6× sequential sprays at ~5-day intervals, with the intent of killing adults emerging from fallen fruiting bodies before they are able to oviposit [11]. Technologies such as transgenic cotton also have no impact on boll weevils [12].

For such reasons and more, practices to delay boll weevil colonization and to restrain population growth are worth pursuing. Two recent non-insecticidal approaches that have shown promise are use of kaolin sprays and removal of potentially infested material [8,13,14]. Kaolin is a common clay mineral containing weathered aluminum silicate; inert, water-soluble, and low in cost, it has been applied in agriculture for various purposes such as plant and fruit protection against heat stress and insect attack. Regarding boll weevil control, kaolin has shown a deterrent effect on feeding and oviposition behaviors [13]. Boll weevil attack rates were lower in experimental fields treated with kaolin [14]. Encouragingly, the same rates of parasitism on caged studies by *Bracon vulgaris* Ashmead, the major boll weevil parasitoid in our areas, and field parasitism rates of this parasitoid and *Catolaccus grandis* Burks were reported under kaolin treatments [10].

We postulated that delayed colonization and restrained population growth might be achieved through a combination of border or whole-field kaolin treatment (depending on field size) and removal of fallen attacked squares from the ground. The aim of field sprays with kaolin would be to cause tactile and visual cue interference, disrupting the arrival of the founding weevils; the removal of attacked fallen squares would take out the potential adult breeders, hence delaying population growth across the season. Thus, in this study we investigated the effects of kaolin sprays on boll weevil oviposition behavior between kaolin-treated and -untreated plants in greenhouse and screened microplot situations upon boll weevil colonization and population growth in small grower areas over two seasons, as compared to both insecticide-treated and untreated controls.

## 2. Experimental Section

The study was conducted in two stages: First, we investigated the boll weevil colonization behavior (feeding and oviposition) using kaolin-treated and untreated plants cultivated in screened greenhouses and caged plants in microplots. These trials were carried out at the experimental area of the “Departamento de Agronomia da Universidade Federal Rural de Pernambuco (UFRPE), Campus Dois Irmãos”, Recife County, State of Pernambuco from December 2010 to March 2011. Second, field colonization and population growth were studied using field plots treated weekly with kaolin in addition to removal of the fallen fruiting bodies. This trial was set up in a grower cotton area located at Surubim County, State of Pernambuco, Brazil (latitude 07°53’48.9’’ S, and longitude 35°49’19.2’’ W) during two cotton growing seasons, 2010 and 2011.

Adult weevils used in the behavior trials emerged in the laboratory from fallen squares collected in cotton fields located at Surubim County, State of Pernambuco. The newly enclosed adults were maintained individually, fed cotton cotyledon leaves for five days, and then paired in plastic vials (50 mL volume) for 48 h to guarantee viable copula before use in the trials.

### 2.1. Boll Weevil Preference towards Kaolin-Treated and -Untreated Plants: Choice Trial

A choice trial was conducted within four anti-aphid screen-protected greenhouses (6 m long × 3 m high × 4 m wide) with arched ceilings and an agricultural plastic film covering. Each greenhouse contained four cylindrical microplots, each of 100 cm diameter × 50 cm high, filled with soil to 45 cm. Three cotton plants of the variety BRS Rubi (brown fiber) were cultivated per microplot, spaced at ~50 cm, for a total of 12 plants per greenhouse and 48 plants overall.

The trial consisted of two treatments: kaolin-treated plants and untreated plants. In each greenhouse, plants in two microplots were sprayed with kaolin, while plants of the other two alternated microplots were not. Each greenhouse composes one replication of treatment. The data of tree plants per microplot and from two microplots per greenhouse constituted a typical replication. Each treatment was evaluated using all 16 microplots. The trial was initiated with plants 65 days after seeding, at which time they were exhibiting the peak of flower buds and young bolls. The average temperature during the trial was 28.3 ± 6.4 °C (mean ± sd) and relative humidity was 54.2% ± 5.81%, measured at 30 min intervals by Datalogger Hobo^®^ (Onset Computer Corp., Bourne, MA, USA).

In each greenhouse, two microplots (six plants) received kaolin and another two (six plants) served as untreated controls. Using a knapsack sprayer (Jacto^®^) with a capacity of 20 L using an empty cone nozzle, we applied Caulisa^®^ kaolin particles (Caulisa, Indústria de Caulim S.A., Campina Grande, Brazil) at a rate of 60 g of kaolin/L of water [13] plus Will Fix^®^ (Charmon Destyl Indústria Química Ltda., Campinas, SP) at 0.05%, which served as surfactant. The next morning between 7 a.m. and 8 a.m. (~16 h after kaolin application), 25 pairs of boll weevils were released into each greenhouse. At 24 h and 48 h after release, each plant was visually inspected for adult boll weevils and damaged fruit bodies (oviposition and feeding punctures).

Prior to statistical analysis, the numbers of weevils and damaged fruit bodies per plant were averaged from the three plants per microplot and two microplots per greenhouse, and were transformed into square root (x + 0.5) to meet the assumptions of normality and homogeneity of the data. The data for kaolin-treated and untreated plants were compared using the paired t-test at 0.05 significance levels (PROC TTEST of SAS; [15]).

### 2.2. Boll Weevil Establishment on Kaolin-Treated and -Untreated Plants: Non-Choice Trial

The non-choice trial consisted of the same cotton plant varieties grown in a manner similar to those for the choice trial, except that planting occurred in 22 outdoor microplots. Eleven caged microplots held kaolin-treated plants (three plants per plot); the other 11 held kaolin-untreated plants. Each microplot was considered as a replication, averaging data from three plants per microplot.

For the kaolin treatment, plants were sprayed at 68 days after seeding, at which time they were exhibiting high densities of flower buds and young bolls (<5 cm diameter). The spray composition and rate were identical to those for the choice test. After being sprayed, three plants were placed in each of 11 100-cm-diameter × 120-cm-high cylindrical cages of anti-aphid screen fastened on iron cylindrical structures; a lateral opening 120-cm long, secured with a 4-cm wide strip of Velcro^®^ allowed access to the interior. Eleven similar cages held trios of unsprayed plants.

At two hours after spraying, two mated pairs of boll weevils were released per cage. At 10 and 20 days after release, we recorded the number of attacked fruiting bodies on the plants and fallen below, and counted the number of living boll weevils. Numbers at 10 days were considered to represent colonization; those recorded at 20 days were considered to denote offspring production. Prior to statistical analysis, numbers of weevils and damaged fruiting bodies per microplot (three plants) were calculated and transformed into square roots (x + 0.5) to meet the assumptions of normality and homogeneity of variance. The comparisons between kaolin-treated and untreated plants were performed using t-tests at 0.05 significance levels (PROC TTEST of SAS; [15]).

### 2.3. Boll Weevil Establishment under Kaolin Treatment in the Field

Field colonization studies were conducted in Furnas, Surubim County, Pernambuco State, Brazil (07°53’48.9’’S, 35°49’19.2’’W) during two crop seasons, with planting dates on April 10, 2010 and May 30, 2011. Using the variety BRS Rubi (brown fiber), cotton was planted in rows 90 cm apart with 5–6 plants per row meter.

Each plot consisted of 10 14-m-long rows of cotton, separated by 5-m strips cultivated with two rows of corn between blocks and treatments. The experimental layout was a randomized block design with four blocks (replications) and two treatments (kaolin-treated plots and kaolin-untreated control plots) during the 2010 season, and three treatments (kaolin-treated plots, insecticide-treated plots, and untreated control plots) during the 2011 season.

Fertilizer at a rate of ~100 g 4:14:8 (N:P:K) per row meter was applied at planting, and 90 g ammonium sulfate (20% N per row meter) was applied at 40 and 80 days after emergence. Weed control was accomplished with a three-tooth horse-drawn cultivator at 30 and 45 days after planting, accompanied by manual hoeing on the row tops as needed. Pesticides were not applied. Temperature and rainfall were monitored from planting to completion of sampling. Temperature was measured at 30-min intervals using a Datalogger HOBO (Onset Computer, Bourne, MA, USA), and rainfall was determined with a pluviometer. Kaolin treatments were applied on a weekly basis at a rate of 60 g of kaolin/L of water [13] plus Will Fix^®^ at 0.05%, which served as surfactant. The volume of kaolin-water dilution applied average from 180 L/ha to 400 L/ha depending on the cotton growth stage. Spraying began 47 days after planting and ended when noticed the first open boll was noticed, for a total of 10 applications in 2010 and 11 applications in 2011.

Insecticide treatment consisted of five applications of 3× organophosphate Methidation (Suprathion 400 EC; Milenia Agrociência S.A., Londrina, Brazil) at rate of 1000-mL ha^−1^ plus 2× pyrethroid Lambda-cyhalothrin (Karate Zeon 50 CS; Syngenta Proteção de Cultivos Ltda, São Paulo, Brazil) at rate of 300-mL ha^−1^. The insecticide was applied at the threshold of 5% damaged squares for crop of up to 55 days old, and a threshold of 10% damaged squares after 55 days [16]. The spray volume of kaolin or insecticides varied from 20L to 40 L per treatment (four blocks) as a function of crop growth stage.

Each week, prior to kaolin or insecticide application, plants were inspected for boll weevil presence and attack on fruiting structures (squares and bolls), and fallen squares were collected. Thus, there were 10 and 11 evaluations during 2010 and 2011 cotton growing seasons, respectively. Ten plants per replication were randomly evaluated by observing three squares and three bolls (when present) per plant, so that 60 fruiting structures were observed per replication and survey date.

During the two years of field studies, the first fallen squares were observed at 62 days after planting (June 11, 2010) and at 53 days after planting (July 22, 2011), and collection of fallen fruiting structures was initiated on these dates. The fallen structures were collected, stored in marked cotton tissue bags, and carried to the laboratory to be evaluated. There, squares and bolls (<10 mm) exhibiting feeding and oviposition punctures were separated from structures that had fallen from other natural causes. After evaluation, samples of ~200 structures (depending on their availability) were kept in 500-mL plastic pots (Prafesta^®^, Mairiporã, Brazil) to allow weevils to emerge. For air flow inside the pots, an opening of 3 cm-diameter was made on the lid and covered with 2 mm nylon mesh; to control moisture, paper toweling was placed at the bottom of the pots and replaced when needed. Temperature was maintained at 25 ± 2 °C, photoperiod at 12 h, and relative humidity at ~60%. The pots were inspected at 5, 10, and 15 days after caging, and the adult weevils that had emerged were recorded and discarded.

To rate cotton production (weight of seeds and fiber) per hectare across treatments, an average was calculated from two subsamples of 1-m rows of plants per replication. The samples were taken at 142 and 145 days after planting during the 2010 and 2011 seasons, respectively. Samples were harvested, placed in identified paper bags, carried to the laboratory, and weighed to the nearest 0.001 g (Kern 430-21).

Numbers of damaged squares and bolls observed in the field were transformed into percentages prior to statistical analysis. For each replication, we rated the number of adult boll weevils observed in the field, the number of fallen structures with oviposition and feeding punctures, the number of emerging boll weevils, and the cotton yield (seeds + fiber) per hectare. Data were subjected to analysis of variance (ANOVA) after being tested for normality (Kolmogorov-Smirnov) and homogeneity of variance (Bartlett’s test), and the data were transformed into square root (x + 0.5) to satisfy the ANOVA assumptions. In addition, an ANOVA (Proc GLM) was conducted using the repeated measure procedure with evaluation dates as a repeated measure (ANOVARM) and two treatments (kaolin-treated and untreated control) during the 2010 season, and tree treatments (kaolin-treated, insecticide-treated and untreated controls) during the 2011 season; while the data from fallen structures (damaged structures and emerged weevils) were subjected to one-way ANOVA. Treatment mean separation was assessed with Tukey HSD tests at a 0.05 significance level. All analyses were performed using the SAS package [15].

## 3. Results

### 3.1. Boll Weevil Preference towards Kaolin-Treated and Untreated Plants: Choice Trial

**Figure 1:**
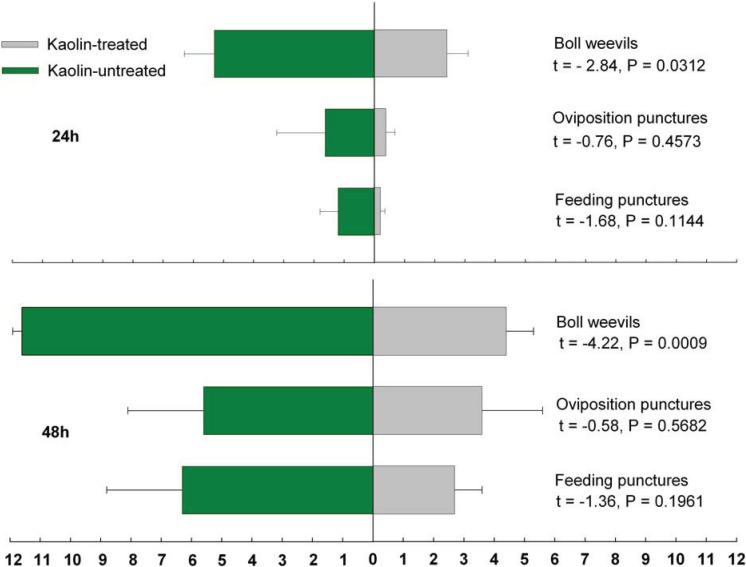
Mean number of boll weevils (*Anthonomus grandis*) recovered, and structures with damage (oviposition and feeding punctures).

Significantly fewer boll weevils colonized kaolin-treated plants as compared to untreated plants, both at 24 h (t_d.f._ = 14 = −2.84, *p* = 0.0131) and at 48 h (t_d.f._ = 14 = −4.22, *p* = 0.0009) after adult release. On average, 2.2× and 2.7× more boll weevils were found on untreated plants at 24 h (5.25 *vs.* 2.37 weevils) and at 48 h (11.8 *vs.* 4.37 weevils), respectively (Figure 1). Likewise, the average numbers of fruiting structures with oviposition punctures were 4.4× and 1.5× higher on untreated plants at 24 h and 48 h from boll weevil release (Figure 1), and the numbers of fruiting structures with feeding punctures were 8.6× and 2.4× greater in untreated plants at 24 h and 48 h from boll weevil releases; however, these numerical differences lacked statistical significance (Figure 1).

### 3.2. Boll Weevil Establishment on Kaolin-Treated and -Untreated Plants: Non-Choice Trial

Kaolin application did not inhibit boll weevil damage under non-choice conditions. Boll weevils caused similar degrees of damage on kaolin-treated and untreated plants as assessed at 10 days (t_d.f._ = 20 = −1.03, *p* = 0.3174) and 20 days (t_d.f._ = 20 = 0.21, *p* = 0.8340) after caging. The average damage (oviposition and feeding) per three plants/microplot at 10 and 20 days after infestation was 10.9 and 38.7 structures for kaolin-treated plants and 9.1 and 36.8 structures for untreated plants, respectively. The numbers of boll weevils alive at 10 days after releasing two pairs per cage, and the numbers of offspring produced 20 days later, were also statistically similar between kaolin-treated (*p* = 0.5725) and untreated (*p* = 0.6657) plants. On average, 0.27 and 0.37 weevils per plant were recovered at 10 days after release, and 18.5 and 23.2 adults emerged 20 days after release.

### 3.3. Boll Weevil Establishment under Kaolin Treatment in the Field

During the 2010 (April–August) and 2011 (May–October) cotton growing seasons, average field temperatures were 23.1 ± 4.7 °C and 22.8 ± 4.5 °C, air relative humidity 49% ± 25.9% and 74.5% ± 8.4%, rainfall of 491 mm and 808 mm, respectively. Regarding the cumulative rainfall during kaolin applications, higher rainfall was recorded during the initial and intermediate kaolin applications in 2010, whereas lower rainfall occurred during 2011 (Table 1).

**Table 1:** Mean temperature (Tm), air relative humidity (RH), and rainfall during the 2010 and 2011 experimental period.

Kaolin sprays	Season 2010 (May 27–August 14)				Season 2011 (June 16–October 2)			
	DAP ^1^	Tm (°C)	RH (%)	Rainfall (mm)	DAP	Tm (°C)	RH (%)	Rainfall (mm)
1st	47	−	−	−	47	−	−	−
2nd	54	26.3	73.6	0	53	21.8	74.5	117
3rd	62	24.9	77.8	48	62	21.5	68.5	101
4th	73	22.8	62.2	280	70	22.8	64.0	27
5th	81	22.6	60.1	28	77	22.2	66.2	0
6th	89	22.4	54.6	5	84	21.9	67.6	2
7th	97	23.1	78.2	10	90	22.0	64.4	60
8th	104	22.0	86.0	4	98	22.8	64.7	3
9th	111	21.9	83.7	5	105	22.4	78.2	0
10th	118	21.4	85.2	10	111	22.6	79.0	2
11th	−	−	−	6	117	23.3	73.9	3
−	−	−	−	−	−	24.8	70.0	0

^1^DAP: days after planting.

**Figure 2:**
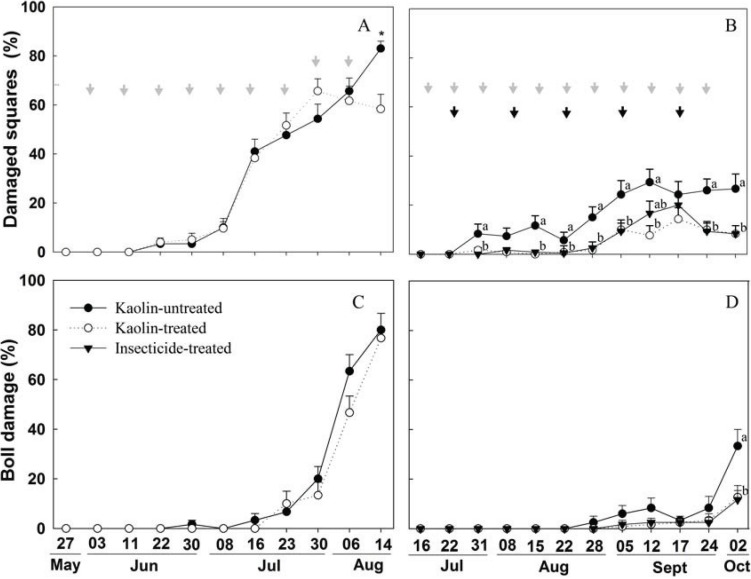
Mean of square and boll damage by boll weevils in kaolin-treated and kaolin-untreated plots during the 2010 season (**A** and **C**), and in kaolin-treated, untreated and insecticide-treated plots during the 2011 season (**B** and **D**). Gray and black arrows indicate kaolin and insecticide applications, respectively. Means (+SE) with different letters for the same date are statistically different at the 0.05 significance level (Tukey HSD’s test).

The first damaged squares observed through plant inspection in the 2010 season occurred at the fourth evaluation (73 days after planting), a time of high precipitation (280 mm). From this evaluation on, the percentage of attack increased progressively during the season (ANOVARM for squares, F_d.f._ = 10, 30 = 110.79, *p* < 0.0001; ANOVARM for bolls, F_10, 30_ = 127.48, *p* < 0.0001) (Figure 2A). A similar progressive rise followed the initial boll weevil attack observations during the 2011 season (squares, F_11, 33_ = 28.79, *p* < 0.0001; bolls, F_11, 33_ = 34.43, *p* < 0.0001) (Figure 2D). During the 2010 cotton-growing season, the average percentage of damaged squares and bolls were similar between kaolin-treated and untreated plots for most of the surveys after verifying boll weevil attack. Only during the last evaluation was the percentage of damage found to be lower in the kaolin-treated plots (F_1, 10_ = 3.33, *p* = 0.0145) (Figure 2A); at this time, damage was 58.3% for kaolin-treated plots, but 83.3% for untreated plots.

During the 2011 cotton season, the first observation of boll weevil damage occurred at the third evaluation (crop 63 days old), at which time damage in insecticide- and kaolin-treated plots (0 and 1.7%) was already lower than in untreated plots (8.3%) (F_2, 119_ = 3.98, *p* = 0.0214) (Figure 2B). The same result—a lower percentage of damaged squares for insecticide- and kaolin-treated plots compared to untreated plots—persisted in the remaining seven evaluations (Figure 2B). Furthermore, boll damage was observed only during the last evaluation and with a lower percentage of damage (F_2, 119_ = 10.42, *p* < 0.0001) in kaolin- and insecticide-treated plots (12% and 11.6%) compared to untreated plots (33.3%) (Figure 2D).

The number of adult boll weevils found per plant varied to a statistically significant degree across surveys during the 2010 season (ANOVARM; F_10, 30_ = 11.79, *p* < 0.0001), but not during the 2011 season (ANOVARM; F_11, 33_ = 1.54, *p* = 0.1096). Despite this population growth along the crop phenology in 2010, there was lack of statistically significant difference between kaolin-treated and untreated plots (F_1, 21_ = 0.02, *p* = 0.8904), with seasonal averages of 0.8 and 0.9 weevils per plant for kaolin-treated and untreated plots, respectively. During the 2011 cotton season, the average number of boll weevils per plant was three-fold greater for untreated plots (0.25) as compared to kaolin-treated plots (0.08), but again there was no statistically significant difference (F_2, 35_ = 1.19, *p* = 0.3495).

The first collections of fallen structures exhibiting boll weevil attack during the 2010 and 2011 cropping seasons occurred when the cotton plants were 73 and 62 days old, respectively. The seasonal averages for numbers of damaged structures collected and boll weevils that emerged were similar across all treatments for 2010 and 2011 seasons (Table 2).

In 2010, the second collection of fallen structures that exhibited boll weevil damage consisted of 35 and 37 structures with oviposition or feeding punctures and resultant emergence of 10 and 5 adults for kaolin-treated and untreated plots, respectively. On the other hand, the second collection of fallen structures during the 2011 season, which also had damaged squares, was very low (Table 2).

Only during the collection seven in 2010 (crop 110 days old), kaolin-treated and kaolin-untreated plots differed significantly in number of damaged fallen structures (F_1, 7_ = 16.04, *p* = 0.0071) and numbers of weevils that emerged (F_1, 7_ = 13.31, *p* = 0.0107). Despite that few statistical differences, the seasonal average was 1,350 fallen structures collected from kaolin-untreated plots and 830 boll weevils subsequently emerging, as compared to 483 fallen structures and 319 boll weevils from kaolin-treated plots (Table 2).

**Table 2:** Mean number of fallen damaged squares and bolls (emerged adult weevils) collected during the 2010 and 2011 cotton-growing seasons in the study plots on family farms in the Semiárido region of Brazil.

Collection	Season 2010			Season 2011			
	DAP ^1^	Untreated	Kaolin-treated	DAP	Untreated	Kaolin-treated	Insecticide-treated
1st	62	0 (0) ^2^	0 (0)	53	0 (0)	0 (0)	0 (0)
2nd	73	35 (10)	37 (5)	62	07 (3)	01 (00)	01 (0)
3rd	81	85 (20)	154 (40)	70	04 (2)	01 (00)	02 (2)
4th	89	258 (96)	241 (87)	77	15 (6)	02 (01)	08 (4)
5th	97	191 (112)	202 (135)	84	09 (5)	02 (01)	02 (1)
6th	104	671 (341)	624 (247)	90	30 (10)	06 (03)	08 (2)
7th	111	1350(830)a ^3^	483 (319) b	98	70 (36) a	12 (09) b	19 (10) b
8th	118	744 (322) a	199 (45) b	105	82 (37)	20 (13)	54 (30)
9th	−	−	−	111	55 (25) a	18 (08) b	38 (23) ab
10th	−	−	−	117	114 (38) a	79 (15) ab	55 (22) b
Totals	−	3,334 (1,731)	1,930 (878)	−	386 (162)	141 (50)	187 (94)

^1^ DAP = Days after planting. ^2^ Fallen structures, but without punctures and weevil emergence. ^3^ Means followed by the same letter within row and season do not differ statistically by Tukey HSD’s test (*p* > 0.05).

The statistical significant differences occurring during the 2011 cotton-growing season started at collection seven (crop 98 days old) and continued thereafter. At the seventh sample interval, the number of damaged fallen structures (F_2, 11_ = 11.49, *p* = 0.0089) and number of emerged boll weevils (F_2, 11_ = 5.31, *p* = 0.0330) differed among kaolin- and insecticide-treated plots in comparison to untreated plots. On average, 12 and 19 damaged structures and 9 and 10 weevils emerged from kaolin- and insecticide-treated plots, respectively, compared to 70 damaged structures and 36 emerged weevils from untreated plots. Further, these statistical differences continued to be significant for collection nine (fallen structures, F_2, 11_ = 5.78, *p* = 0.0399; boll weevil emergence, F_2, 11_ = 36.84, *p* = 0.0004) and 10 (fallen structures, F_2, 11_ = 5.73, *p* = 0.0406; adult emergence, F_2, 11_ = 13.40, *p* = 0.0061) (Table 2).

Despite the lack of difference demonstrated across each evaluation, the overall seasonal reduction in damage was about 50% (fallen structures and emergence of boll weevils) during 2010 season in kaolin-treated (1,930 structures and 878 weevils) compared to untreated plots (3,334 structures and 1,731 weevils). Approximately the same seasonal reduction in damage was observed for the 2011 season and per date of evaluation for kaolin- and insecticide-treated plots in comparison to untreated plots (Table 2).

**Figure 3:**
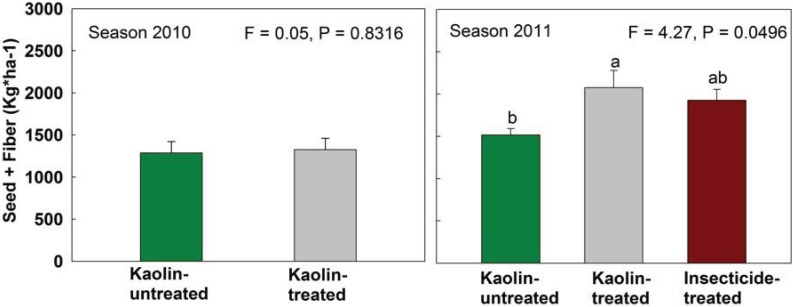
Cotton yield (seeds + fiber) from kaolin-untreated and kaolin-treated 2010 season, and kaolin-untreated, kaolin-treated and insecticide-treated 2011 season. Bars (+SE) under the same letters do not differ statistically by Tukey HSD’s test (*p* > 0.05).

The cotton production yields per hectare measured for kaolin-treated and -untreated plots in 2010 were similar (F_1, 7_ = 0.05, *p* = 0.8316, Figure 3). On the other hand, superior yield was found for kaolin- and insecticide-treated plots in the 2011 season (F_2, 11_ = 4.27, *p* = 0.46), as compared to untreated plots (Figure 3).

## 4. Discussion

Kaolin reduced boll weevil colonization of cotton plants when beetles were presented with a choice between kaolin-treated and untreated plants, but not under non-choice conditions. After colonization, kaolin application did not restrain damage and population growth under the confined conditions in microplots or in the field when heavy rainfall occurred during the field colonization period.

It seems reasonable to postulate that these observations reflect the operation of different cues at different stages in boll weevil host recognition/colonization behaviors. During the initial stages, the brilliant white of the dried kaolin film may hinder visually-based substrate localization in a manner analogous to the protection obtained with reddish leaf cotton varieties, which are subject to less oviposition [17], as we found in the choice trial, especially in the 24 h-evaluation period (Figure 1, 24 h). Later, after boll weevils are on the plant, other cues probably come into play, and feeding and oviposition rates are similar to those for untreated plants as we found in the 48 h-evaluation in the choice trial (Figure 1, 48 h) and in the non-choice trial. Our results showed reduction of kaolin efficacy in restraining damage over time even during the choice trial. The difference ratio of damaged structures between kaolin-treated and untreated plants dropped from 4.4× to 1.5× between 24 h and 48 h evaluations (Figure 1). This corroborates the non-choice trial results in which similar damage was recorded from kaolin-treated and untreated plants.

An array of studies have used kaolin against other cotton pests such as beet armyworm, *Spodoptera exigua* (Hübner) [18], pink bollworm, *Pectinophora gossypiella* (Saunders) [19], old world cotton bollworm *Helicoverpa armigera* (Hübner) [20] and cotton aphids *Aphis gossypii* Glover [21,22]. The fact that these studies have produced varying results may be a reflection of the species’ different morphology, feeding and oviposition habits. For example, the boll weevil has a rostrum-like beak with chewing mouthparts located at the tip of the rostrum; this allows the adult boll weevil to puncture the square and boll for feeding and oviposition internally in a manner quite different from Lepidopteran larvae, which chew and ingest the whole treated surface. Additional behavioral differences such as low locomotion of boll weevil and localized feeding exhibited by boll weevil adults also may have weakened any inhibitory feeding and oviposition effects of kaolin for this species after plant colonization.

Kaolin application inhibits host choice for many other cotton pest species that feed on fruiting structures or leaves. Lower populations of beet armyworm larvae [18], and lower oviposition by old world bollworms were reported on kaolin-treated cotton [20]. The combination of kaolin and lambda-cyhalothrin promoted efficacious control of pink bollworms, and when they were applied alone, kaolin performed better than lambda-cyhalothirn [19]. Likewise, damage by stinkbugs was lower on cotton treated with kaolin [23]. On the other hand, the effect of kaolin on cotton aphids, which like boll weevils are puncture feeders, has been variable. Higher aphid populations on ventral leaf surfaces of kaolin-treated cotton [21], whereas after testing solutions with 2%–8% of kaolin, a solution with 5% kaolin was recommended [22] for field trials aimed at cotton aphid control.

Precipitation patterns can have a major impact on the efficacy of kaolin spraying, as demonstrated by our different results for the 2010 and 2011 growing seasons (Table 1). In 2010, high rainfall diminished the efficacy of kaolin applications, resulting in no detectable statistical differences between treatments. The opposite occurred during the 2011 season; kaolin-treated plots exhibited lower infestation and boll weevil damage from the first evaluation, and kaolin treatment resulted in yields that were higher than those for untreated plots and similar to yields for insecticide-treated plots (Figure 3). It is important to highlight, however, that the overall boll weevil population was lower in the 2011 season compared to the 2010 season (Figure 2). Rainfall was responsible for low efficacy of kaolin protection in a previous field trial [13]. Thus, it seems clear that when periods of high precipitation coincide with boll weevil colonization, the kaolin film that is washed away by the rain should be reapplied for continued effectiveness.

The emergence in the laboratory of large number of boll weevils from collected abscised structures during the 2010 season from both kaolin-treated and untreated plots indicates the positive impact of collecting fallen structures. Despite early colonization by boll weevils, the number of damaged fallen squares was greater at the end of the season in the control untreated plots. Among the collected structures, most were squares exhibiting boll weevil oviposition punctures. Squares abscission is caused by weevil oviposition, but newly formed bolls (<5 mm) with oviposition and squares with feeding punctures may also fall from other causes, especially in the Semiárido conditions where the plants suffer from nutrient and water deficits [8].

Collecting fallen structures, especially early in the season, is of crucial importance to the success of the management practice presented herein. It aims to reduce the first generation of boll weevils inside the field [8,24]. In both 2010 and 2011, the collection of fallen structures carried out in this study removed a substantial number of boll weevils (Table 2) that otherwise might have emerged and enlarged subsequent generations. Moreover, collection of fallen structures combined with kaolin sprays delayed boll weevil population growth in 2011 compared to untreated plots. The first collection of damaged fallen structures occurred when the crop was 77 days old, at which time there were already fewer damaged structures from kaolin-treated plots compared to untreated plots.

Cotton production was higher in 2011 than in 2010, particularly in the kaolin- and insecticide-treated plots. Beyond lower boll weevil populations observed in 2011 season, the rainfall in 2010 was very low early in the season (Table 1); this compromised plant development and early square production as compared to the 2011 season. Beyond that, the return of rainfall in 2010 occurred when crops were 62–81 days old, reducing the protection offered by kaolin application during the period of boll weevil colonization. This resulted in greater damage by boll weevils (Table 2). Furthermore, in the 2011 season, the rainfall dropped after the crop was 70 days old, allowing the maintenance of the kaolin film on plants affecting boll weevil colonization; and further population growth through collection of fallen structures that removed weevils from first generation (Figure 2B). In addition, the clipping terminals of plants with 50% open bolls at the end of the season to remove the non-reproductive squares and bolls also promises to have a direct impact on boll weevil populations; in cage trials, collecting abscised reproductive structures, clipping plant terminals, and using both practices together reduced boll weevil populations by as much as 63%, 57%, and 79%, respectively [8]. Using the two tested practices and the clipping plant terminals together could provide a feasible way to significantly impact boll weevil populations, especially for smallholder cotton growers.

## 5. Conclusion

The confinement and field trials allow us to infer that kaolin application interfered with boll weevil colonization as expected, but did not restrain feeding and oviposition behaviors when the weevil was on the plants. To ensure the plant is continuously camouflaged from boll weevils by using kaolin, reapplication right after rainfall is necessary, especially during critical crop phenology matching with boll weevil colonization in the area. Kaolin application at the proper time to reduce field colonization and removal of fallen structures, especially those early in season that are caused by founding females, are two non-chemical practices that could be of crucial importance to manage boll weevils, particularly for cotton grown on family farms in the Semiárido region. Additional cultural control practices such as clipping the terminals of plants with 50% open bolls at the end of the season to remove the non-reproductive squares and bolls—which are all no-chemical practices and of low cost—could be applied together as a feasible way to significantly impact boll weevil populations, especially for smallholder cotton growers.
